# Mitochondrial Cardiomyopathy: The Roles of mt-tRNA Mutations

**DOI:** 10.3390/jcm11216431

**Published:** 2022-10-30

**Authors:** Yu Ding, Beibei Gao, Jinyu Huang

**Affiliations:** 1 Central Laboratory, Hangzhou First People’s Hospital, Zhejiang University School of Medicine, Hangzhou 310036, China; 2 Department of Cardiology, Hangzhou First People’s Hospital, Zhejiang University School of Medicine, Hangzhou 310006, China

**Keywords:** cardiomyopathy, mt-tRNA, mutations, OXPHOS system, tRNA biology

## Abstract

Mitochondria are important organelles whose primary role is generating energy through the oxidative phosphorylation (OXPHOS) system. Cardiomyopathy, a common clinical disorder, is frequently associated with pathogenic mutations in nuclear and mitochondrial genes. To date, a growing number of nuclear gene mutations have been linked with cardiomyopathy; however, knowledge about mitochondrial tRNAs (mt-tRNAs) mutations in this disease remain inadequately understood. In fact, defects in mt-tRNA metabolism caused by pathogenic mutations may influence the functioning of the OXPHOS complexes, thereby impairing mitochondrial translation, which plays a critical role in the predisposition of this disease. In this review, we summarize some basic knowledge about tRNA biology, including its structure and function relations, modification, CCA-addition, and tRNA import into mitochondria. Furthermore, a variety of molecular mechanisms underlying tRNA mutations that cause mitochondrial dysfunctions are also discussed in this article.

## 1. Introduction

The term “cardiomyopathy” refers to a condition in which the heart muscle is abnormal in thickness, stiffness, or strength. There are several types of cardiomyopathy, named dilated cardiomyopathy (DCM), hypertrophic cardiomyopathy (HCM), restrictive cardiomyopathy (RCM), and arrhythmogenic right ventricular cardiomyopathy (ARVC) [1,2]. Among them, DCM is the leading cause of heart failure (HF) [3], while HCM is a frequent genetic disease associated with nuclear or mitochondrial gene mutations. RCM is a mix of diseases featured by stiffness of the ventricular walls, which finally leads to HF [1]. ARVC is a pathological condition linked to the replacement of cardiac with fibrofatty tissues, which results in reduced cardiac functions and increases the risk of sudden cardiac death [1]. In fact, primary cardiomyopathy can be genetic, acquired, or mixed in etiology [4]. In particular, genetic cardiomyopathies are caused by chromosomal abnormalities that affect the heart [5]. Despite the fact that the etiology for these cardiomyopathies is different, there is an obvious inherited factor that contributes to the progression of this disease. It is well-recognized that autosomal recessive, X-linked, and matrilineal inheritance are the main patterns for cardiomyopathy [6,7]. Because mtDNA generates more than 90% of ATP, which is essential for normal heart functions [8], defects in mitochondrial function have been regarded as an important contributor to cardiomyopathy [9].

**Figure 1:**
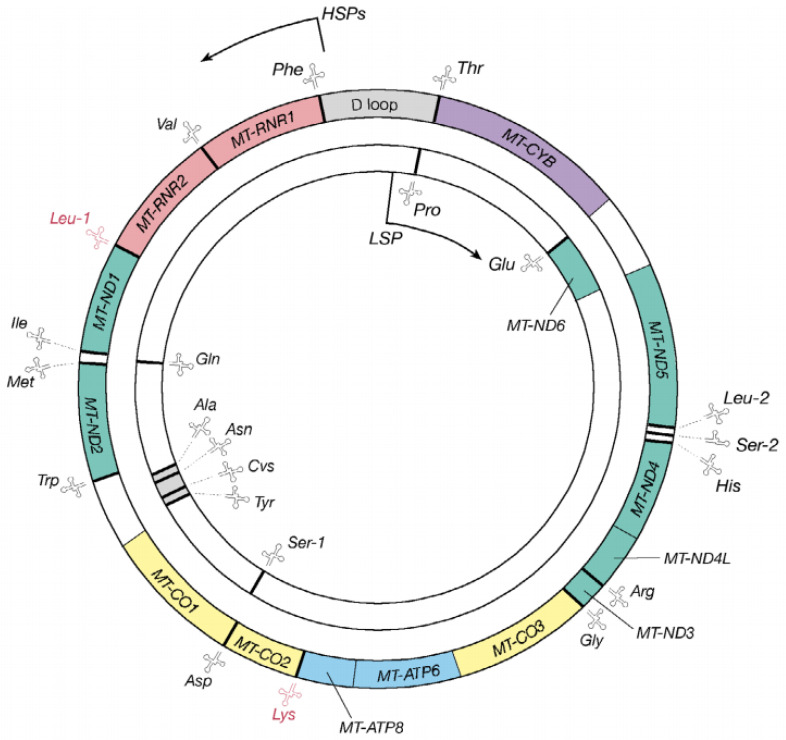
The genetic map of the human mitochondrial genome, which is 16,569-bp. The outer circle presents the heavy strand, while the inner circle indicates the light strand and 22 genes encoding mt-tRNA molecules are distributed throughout the mtDNA genome.

Human mitochondria are membrane-bound cell organelles that play important roles in regulating programmed cell death or necrosis, affecting cellular proliferation and metabolism, and promoting cholesterol synthesis [10]. However, the most important function of these organelles is to generate ATP via OXPHOS and release reactive oxygen species (ROS) as a toxic byproduct [11]. In fact, as shown in Figure 1, human mitochondrial DNA (mtDNA) is a relatively small (16,569-bp), double-strand molecule that contains 13 genes for peptides for mitochondrial respiratory chain (MRC), two for mitochondrial rRNAs (12S rRNA and 16S rRNA) and 22 for mt-tRNA [12]. To date, more than 200 pathogenic mtDNA mutations have been mapped into mt-tRNA genes (http://www.mitomap.org/MITOMAP, accessed on 15 August 2022) [13], emphasizing the importance of mt-tRNAs for mitochondrial function [14]. In the current review, we provide an overview of the recent progress on human mt-tRNAs and discuss the potential mechanisms underlying cardiomyopathy-associated mt-tRNA mutations.

## 2. mt-tRNA Genes and Structure

**Figure 2:**
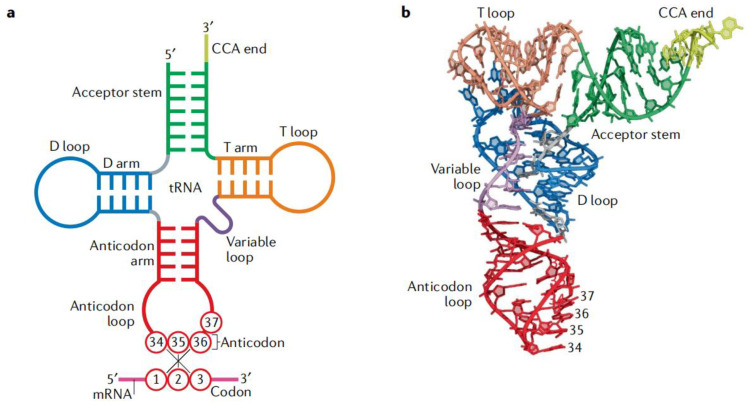
Basic structure and function of tRNA. (**a**) Cloverleaf structure of tRNA with codon–anticodon pairing: the tRNA consists of five parts: Acceptor arm, D-arm, anticodon stem, Variable region, and TΨC loop. (**b**) Tertiary structure of tRNA: the coordinates are obtained from Protein Data Bank entry 1EHZ. The color code is the same as in part a.

As the adaptor that decodes the mRNA sequence into protein, the basic aspects of mt-tRNA structure and function are central to all studies of mitochondrial biomedicine. Almost every mt-tRNA has a cloverleaf structure consisting of an Acceptor arm, D-arm, anticodon stem, Variable region, and TψC loop, with an average length of approximately 73-bp [15]. Of 22 mt-tRNAs, MT-TE, MT-TA, MT-TN, MT-TC, MT-TY, MT-TS1, MT-TQ, and MT-TP occur at the L-strand, the rest, MT-TF, MT-TV, MT-TL1, MT-TL2, MT-TI, MT-TM, MT-TS2, MT-TW, MT-TD, MT-TK, MT-TG, MT-TR, MT-TH, and MT-TT, are present in the H-strand [16]. Interestingly, the tRNA cloverleaf structure forms an interaction between the D-arm and the TΨC loop, while the anticodon stem, which spans the positions of 34 to 36 of the canonical tRNAs, is the place where the codon and anticodon interact [17] (Figure 2).

Intriguingly, the secondary structure of the tRNA^Ser(AGY)^ lacks the entire D-arm [18], which is common in various mammalian mitochondrial genes. Remarkably, human mt-tRNA^Ser(UCN)^ has some special characteristics: only one base spanning the Acceptor arm and D-arm, as well as a shortened D-arm and an extra loop [19].

## 3. mt-tRNA 5′ and 3′ End Processing

mt-tRNAs require essential maturation steps to become functional. These maturations comprise endoribonucleolytic and/or trimming of 5′ and 3′ extensions, tRNA splicing, base modifications, base editing, and CCA addition that allows aminoacylation [20]. In particular, the RNase P, which was first identified in bacteria, is responsible for 5′ end maturation [21]. Human mitochondrial RNase P (mt-RNase P) consists of three protein sub-units: TRMT10C, SDR5C1, and PRORP, all of which are encoded by the nuclear DNA (nDNA) [22,23].

3′-end processing pathways, by contrast, are more diverse. This biochemical process is performed by the endonuclease RNase Z or the exonuclease Rex1p [24]. Compared with RNase P, RNase Z is very conserved in various species and belongs to the β-lactamase superfamily [25]. In addition, RNase Z has two isoforms; a smaller form, named RNase ZS, has been identified in the three domains of life (Archaea, Bacteria, and Eukaryote), whereas the other version, called RNase ZL, is an enzyme that is much larger than RNase ZS and found only in eukaryotes [26,27].

## 4. mt-tRNA Chemical Modification

After transcription by RNA polymerase, tRNA precursors usually undergo post-transcriptional processing, including numerous bases or sugar modifications, by various tRNA modifying enzymes [28]. These chemical modifications are critical for the stabilization of tRNA structure, allowing for proper interactions with other molecules and protection of tRNA from degradation [29]. The development of mass spectrometry has allowed us to provide an accurate way to identify unknown chemical modifications. To date, approximately 28 modified nucleosides spanning 46 positions have been found in human mt-tRNAs (Table 1) [30], and many of these modifications are broadly conserved in bacteria, eukaryotes, and archaea [31]. Among these, 15 modifications are called “universal modifications” since they are present in three domains of life [32] and mitochondria-specific residues at the wobble position 34. Actually, to maintain its normal function, position 34 is required for two taurine-associated modifications in mt-tRNAs: τm^5^U for tRNA^Leu(UUR)^ and tRNA^Trp^ and τm^5^s^2^U for tRNA^Glu^, tRNA^Lys^, and tRNA^Gln^ [33]. These modifications are essential for accurate protein translation, as well as codon and anticodon interactions [33]. For instance, the lack of τm^5^U34 modifications caused by diabetes-associated tRNA^Leu(UUR)^ A3243G mutation is responsible for translational deficiency [34].

Another important region for chemical modification is located at the anticodon stem, just 3′ to the anticodon [28]; modification at position 37 would keep the A-site anticodon functions and promote the accurate translational reading frame. By contrast, disease-associated mtDNA mutations such as tRNA^Met^ A4435G, which disrupts position 37 modifications, would decrease the tRNA steady-state level and affect its functions [35].

## 5. tRNA Aminoacylation

The aminoacyl-tRNAs (aa-tRNAs), which are first catalyzed by aminoacyl-tRNA synthetases (aaRSs) and then delivered to a macromolecular called ribosome, play critical roles in efficient protein synthesis [36]. In particular, mitochondrial aminoacyl-tRNA synthetases (mt-aaRSs) are encoded by nDNA, ensure the proper attachment of each amino acid (AA) to its cognate mt-tRNA, and are imported into mitochondria from the cytoplasm [37]. There are two steps for universal aminoacylation reaction: (1) the mt-aaRS binds an AA with ATP, thus creating aminoacyladenylate and pyrophosphate; (2) the AA residue is brought proximal to the 3′ end of a specific mt-tRNA [38].

In humans, 17 mt-aaRSs are responsible for 20 standard AAs [39]. Genes encoding these proteins are designed as ARS2: for instance, AARS2 is referred to as the alanyl-tRNA synthetase. However, the GARS, which stands for glycyl-tRNA synthetase, encodes both cytosolic and mitochondrial proteins [40]; in addition, the KARS, which is responsible for lysyl-tRNA synthetase, employs splicing to form distinct mRNAs [41].

Theoretically, mutations in mt-aaRSs that impaired the maturations of mt-tRNAs were believed to have functional consequences for protein synthesis [42]. Recent experimental studies revealed that mt-aaRSs mutations predominantly affected the central nervous system (CNS) [43]. In the CNS-related pathologies, mutations in eight mt-aaRSs, including *RARS2*, *NARS2*, *CARS2*, *IARS2*, *FARS2*, *PARS2*, *TARS2,* and *VARS2* led to mitochondrial myopathy, four mt-aaRSs, *AARS2*, *DARS2*, *EARS2,* and *MARS2* mutations caused the leukodystrophies, and two mt-aaRSs, *HARS2* and *LARS2* mutations were involved in Perrault syndrome [44,45,46].

## 6. 3′ End CCA Addition

Functional mt-tRNA maturations require the 3′ end CCA addition [47]. In most organisms, this essential sequence is not encoded in the tRNA genes. Instead, this process is under the control of a CCA-adding enzyme called tRNA nucleotidyltransferase [48]. In *homo sapiens*, this gene is named TRNA-Nucleotidyltransferase 1 (*TRNT1*). There are 7 exons in this gene, which is localized at 3p26.2 and spans about 20-kb in length [49]. 

*TRNT1* is a protein-coding gene. This essential enzyme functions by catalyzing the addition of the conserved nucleotide triplet CCA to the 3′ end of tRNA molecules [50]. Several steps must be tightly coordinated by the *TRNT1* to ensure error-free CCA addition. To begin with, *TRNT1* must identify tRNA and tRNA-like substrates, use only CTP and ATP, but exclude UTP and GTP, and switch specificity from C to A nucleotide after adding CC nucleotides and stop polymerization. Notably, in the absence of any of these steps, a tRNA molecule cannot be charged with an AA or perform any translational function [51]. 

Interestingly, there have recently been reports of mutations in the *TRNT1* that reduced its catalytic activity, resulting in congenital sideroblastic anemia with immunodeficiency, fevers, and developmental delay (SIFD) [52,53]. Furthermore, mitochondrial translation may be impaired by mt-tRNA^Ser(AGY)^ CCA addition associated with *TRNT1* mutations, leading to a decrease in OXPHOS complexes abundance [54,55]. 

## 7. Import of tRNAs into Mitochondria

Most mitochondrial proteins are encoded by nuclear genomes and thus have to be imported into mitochondria from the cytosol. Furthermore, as the number of tRNA genes is insufficient for proper protein synthesis according to the genetic code and on the wobble rules, this lack of nuclear tRNAs could be compensated by the import of nuclear-encoded tRNAs [56]. About 40 years after its discovery in *Tetrahymena pyriformis* [57], tRNA import was recognized as a vital step in mitochondrial biogenesis [58,59]. In general, the import of tRNAs from the nucleus to the mitochondria consists of two key steps: the first is the targeting of tRNAs to the mitochondria; the second process involves their translocation via the mitochondrial membranes to reach the matrix [60,61]. 

In mammalian mitochondria, RNA import occurs through two different mechanisms: one involves cytosolic factors and an intact protein import system, while the other does not require soluble factors [62]. According to the first one, tRNAs are imported along the protein import pathway in a complex with a mitochondrial precursor. Initial studies were conducted in yeast, where tRNA^Lys^ was co-imported with the pre-LysRS [63]. The second mechanism was characterized by the direct importation of tRNAs into isolated mitochondria without cytosolic factors; a case in point was the import of tRNA^Gln^ into mitochondria [64]. 

Mt-tRNA mutations cause respiratory deficiencies and lead to a wide range of mitochondrial disorders. Many of these mutations have unclear molecular consequences, and there are no effective treatments. However, the concept of mitochondrial tRNA import presents a novel treatment opinion; if a cytosolic tRNA were injected into the mitochondria that were capable of replacing the mutant mt-tRNA, it would be of great significance. A recent experimental study confirmed this hypothesis and found that in cybrid cells bearing myoclonic epilepsy with ragged-red fibers (MERRF)-associated tRNA^Lys^ A8344G mutation, in addition to restoring tRNA^Lys^ function, mitochondrial translation, complex respiratory activity, and other functions were partially rescued after import of tRNA^Lys^ [65]. Thus, the use of tRNA import could be a novel strategy to cure mitochondrial disorders [66,67]. 

## 8. Cardiomyopathy-Associated mt-tRNA Mutations

### 8.1. tRNA^Phe^ Mutation

The homoplasmic tRNA^Phe^ T593C mutation was identified in patients with optic neuropathy, cardiomyopathy, and cognitive disability [68]. In human mitochondrial databases, such as mtDB (http://www.mtdb.igp.uu.se/, accessed on 15 August 2022) or Mitomap (http://www.mitomap.org/MITOMAP, accessed on 15 August 2022), this mutation was reported to be a rare polymorphism in the general population [69]. However, it may affect the progression of Leber’s Hereditary Optic Neuropathy (LHON) and non-syndromic hearing impairment in Asian populations [70,71]. Analysis of muscle biopsy samples revealed reduced values for oxygraphic *Vmax* of complexes I + III + IV, and that the respiratory chain complexes (RCC) I, III, and IV experienced a severe decrease in activity, highlighting the contribution of m.T593C mutation to mitochondrial dysfunction.

### 8.2. tRNA^Val^ Mutations

The m.C1628T and m.G1644A mutations were identified in two Spanish patients with cardiomyopathy. Examination of muscle biopsy showed combined deficiencies of RCC I and IV [72]. Moreover, the m.C1628T or m.G1644A mutation markedly affected the steady-state level of tRNA^Val^, suggesting that these mutations can cause tRNA metabolism failure and contribute to cardiomyopathy [72].

### 8.3. tRNA^Leu(UUR)^ Mutations

A hot spot for pathogenic mutations associated with cardiomyopathy is mt-tRNA^Leu(UUR)^, including m.A3243G [73,74,75], m.T3250C [76], m.A3260G [77,78], m.T3271C [79], and m.C3303T [80,81] mutations. The well-known m.A3243G mutation is one of the most important causes of cardiomyopathy. In fact, the A-to-G transition at 3243 of mtDNA was reported to be the most prevalent mutation for various mitochondrial diseases such as diabetes [82], mitochondrial encephalomyopathy with lactic acidosis and stroke-like episodes (MELAS) [83], MERRF [84], and maternally transmitted diabetes and deafness (MIDD) [85]. Since mitochondrial disease is a multisystem presentation, the examination of skeletal muscle pathology was recommended for the diagnosis of mitochondrial cardiomyopathy [86].

In addition to the inefficient aminoacylation of tRNA^Leu(UUR)^ [87], m.A3243G also altered the mitochondrial RNA precursors, as well as its base modification [88]. Cybrids containing the m.A3243G mutation exhibited a 70–75% reduction in aminoacylated tRNA^Leu(UUR)^, contributing to a shortage of this tRNA, thus leading to defects in protein synthesis [89,90,91].

The m.T3250C mutation was described in patients harboring lactic acidosis, chronic fatigue, exercise intolerance, and muscle weakness [92,93]. Patient-derived fibroblast cell lines confirmed that this mutation affected mitochondrial function, evidenced by a lower level of ATP and RCC actives and a higher amount of ROS [76]. Thus, the m.T3250C mutation affected mitochondrial respiration and resulted in cardiomyopathy through incomplete penetrance. 

The A-to-G transition at position 3260 was listed on the Mitomap database (http://www.mitomap.org/MITOMAP, accessed on 15 August 2022) as a confirmed mutation associated with maternal myopathy and cardiomyopathy [94,95]. In cybrid cells harboring the m.A3260G mutation, as compared to controls without this mutation, the rate of oxygen consumption, RCCs activities, and lactate production were markedly abnormal [77]. Furthermore, the m.A3260G mutation affected the respiratory chain functions and caused defects in the OXPHOS system [77]. 

Patients with MELAS-like syndrome, as well as diabetes, were traditionally reported to have the m.T3271C mutation [96,97,98]. Subjects with m.T3271C mutation exhibited a marked decrease in RCCs I + IV activities [99]; moreover, defects of τm^5^U modification at the anticodon wobble position caused by this mutation, aggravated the tRNA^Leu(UUR)^ metabolism failure, thereby resulting in mitochondrial dysfunction [100].

The heteroplasmic mutation m.C3303T was originally reported in a pedigree carrying cardiomyopathy and myopathy [101]. This mutation abolished the conserved base-pairing in the Acceptor arm of tRNA^Leu(UUR)^; in addition, there was a biochemical defect with RCCs I~IV, indicating that m.C3303T mutation was responsible for the impairment of mitochondrial protein translation [102,103]. 

### 8.4. tRNA^Ile^ Mutations

The homoplasmic m.T4277C mutation occurring in the D-arm of tRNA^Ile^ was identified in a patient with HCM and hearing impairment [104]. Skeletal muscle showed multiple changes in respiratory chain enzymes and a lower steady-state level of tRNA^Ile^ with m.T4277C mutation. Notably, approximately 70% reduction in tRNA^Ile^ steady-state level was observed in the skeletal muscle of the patients with this mutation, which is below the threshold for normal cell function, resulting in the clinical phenotype [104].

The heteroplasmic m.A4295G mutation is located directly 3′ end immediately to the anticodon stem of the tRNA^Ile^, which is very conserved in various species [105]. Notably, the m.A4295G mutation introduced an m^1^G37 modification of tRNA^Ile,^ which was catalyzed by methyltransferase 5 (TRMT5) [106]. Simulations of molecular dynamics suggested that the m.A4295G mutation altered the structure and function of tRNA^Ile^, as evidenced by enhanced *Tm*, structural alternations, and instability of mutated tRNA. Using in vitro processing experiments, the m.A4295G mutation was found to reduce the tRNA^Ile^ 5′ end processing efficiency [107]. Therefore, the m.A4295G mutation may affect the OXPHOS system and lead to mitochondrial dysfunction.

The m.A4300G in tRNA^Ile^ is regarded as a pathogenic mutation for maternally inherited cardiomyopathy [108]. Molecular and biochemical analysis suggested that the m.A4300G mutation significantly decreased the RCCs, as compared with the controls without this mutation [109]. Furthermore, Northern blot analysis demonstrated that the m.A4300G caused ~45% reductions in steady-state levels in tRNA^Ile^ [109].

The m.A4317G mutation in tRNA^Ile^ affected tRNA by forming an abnormal stable structure in the TψC loop, thus increasing the *Tm* value [110]. The changes in secondary structure can influence the tRNA^Ile^ maturations, such as CCA addition in the 3′end [111]. Moreover, the m.A4317G mutation was reported to decrease isoleucylation significantly and was involved in the pathogenesis of fatal infantile cardiomyopathy [112]. 

Interestingly, an m.4322dupC mutation in the tRNA^Ile^ gene was reported to be associated with DCM. This insertion was present heteroplasmic in blood and muscle. Biochemical analysis showed that the m.4322dupC reduced levels of RCC activities [113].

### 8.5. tRNA^Trp^ Mutation

The m.G5521A mutation, as well as the *CO2* G8249A mutation, was reported in Tunisian patients with cardiomyopathy [114]. The m.G5521A mutation occurred at the D-arm of tRNA^Trp^, which might disrupt the secondary structure and functions of this tRNA, thereby causing a reduction in mitochondrial protein synthesis [101].

### 8.6. tRNA^Cys^ Mutation

The homoplasmic m.A5814G mutation was first reported in an infant manifesting DCM, MELAS [115]. The m.A5814G mutation may affect the secondary structure of the tRNA^Cys^ gene, altering the highly conserved last pairing of the D-arm region [116]. Interestingly, the tRNA^Leu(UUR)^ A3252G, which occurred at the same position as the m.A5814G, was regarded as a pathogenic mutation for MELAS-like syndrome [117]. Therefore, the m.A5814G mutation may have the same impact on tRNA translation and lead to the impairment of mitochondrial function.

### 8.7. tRNA^Ser(UCN)^ Mutation

The homoplasmic m.A7495G mutation abolished a very conserved Watson–Crick base-pairing in the D-arm of tRNA^Ser(UCN)^. Mutation at that position was critical for mt-tRNA structure and function. Moreover, a significant decrease in COX and Complex I activities was observed as compared to controls [118], indicating that this mutation may affect OXPHOS function.

### 8.8. tRNA^Lys^ Mutations

The heteroplasmic m.T8306C mutation in the tRNA^Lys^ gene was reported in a patient with severe late-onset of myopathy, myoclonus, leukoencephalopathy, HCM, and metabolic syndrome [119]. This change disrupted a T-A bond in the D-arm of tRNA, a nucleotide that was well conserved via evolution and is likely to have functional importance. Biochemical analysis of complex activities revealed a multiple defect in RCCs (I + III + IV), and single fiber analysis demonstrated that this mutation segregated with COX-deficient fibers [120]. 

The well-known m.A8344G mutation is commonly associated with MERRF [121,122]. In addition, this mutation is associated with cardiomyopathy based on a recent study [123]. Using cybrid cells with this mutation, the m.A8344G mutation was found to cause a defect in τm^5^s^2^U modification [124]. Importantly, tRNA^Lys,^ without this modification, was unable to translate its genetic codons (AAA or AAG) because of the complete loss of codon and anticodon interactions on the ribosome [125]. Thus, the lack of wobble modification caused by m.A8343G mutation led to a translational defect, contributing to mitochondrial dysfunction [126]. 

In addition, the heteroplasmic m.G8363A was first described in a US family with inherited cardiomyopathy and hearing impairment [127]. The m.G8363A mutation abolished the conserved base-pairing in the Acceptor arm of tRNA^Lys^ and may affect the tRNA structure and function. Single-fiber PCR analysis suggested a significant link between mutant mtDNA and impaired biochemical activities [128]. Moreover, the m.G8363A mutation caused a marked reduction in its aminoacylation ability, suggesting that this mutation was definitely pathogenic for cardiomyopathy [129]. 

### 8.9. tRNA^Gly^ Mutation

The heteroplasmic tRNA^Gly^ T9997C mutation was reported in a multiplex family manifesting non-obstructive cardiomyopathy [130]. This mutation affected the position adjacent to the Acceptor arm of tRNA^Gly^. The m.T9997C is very conserved in invertebrates and mammals. Functional analysis indicated that the m.T9997C mutation reduced the activities of RCCs and protein synthesis [131]. 

### 8.10. tRNA^His^ Mutation

The m.G12192A mutation was originally reported in a Japanese patient who had reduced contraction of the left ventricle [132]. Furthermore, the co-occurrence of m.G12192A and m.G11778A mutations was detected in subjects with LHON and cardiomyopathy [133]. It is interesting to note that the m.G12192A mutation occurred at the TψC loop of tRNA^His^, which was conserved from different vertebrates; in addition, a significant reduction in ATP and enhanced ROS levels were found in cell lines derived from patients carrying this mutation [134], emphasizing the contributions of m.G12192A mutation to mitochondrial dysfunction.

### 8.11. tRNA^Leu(CUN)^ Mutation

The mitochondrial heteroplasmic m.T12297C mutation affecting a highly conserved nucleotide (adjacent to the anticodon triple) was reported in an Italian family with cardiomyopathy and endocardial fibroelastosis [135]. Interestingly, the m.T12297C mutation played an important role in the interactions between mRNA and anticodon; therefore, mutant tRNA^Leu(CUN)^ was less stable than the wild-type version of this tRNA [136]. Patients harboring this mutation exhibited a significant reduction in RCC I activity, suggesting a positive link between this mutation and cardiomyopathy [137].

### 8.12. tRNA^Glu^ Mutation

The m.T14709C affecting a conserved position in the anticodon stem of tRNA^Glu^ has been described in patients with diabetes and myopathy [138,139,140]. Functional analysis using blue native PAGE showed an increased mtDNA content and decreased RCC activities, suggesting that the m.T14709C mutation was pathogenic for this disease [141].

### 8.13. tRNA^Thr^ Mutation

The m.A15924G mutation occurs at the extremely conserved nucleotide of tRNA^Thr^, which is the last base pair of the anticodon stem adjacent to the anticodon loop of this tRNA [16]. Interestingly, the m.A15924G mutation abolished the Watson–Crick base-pairing and may result in the failure in tRNA metabolism. Functional assessment of DCM patients with m.A15924G mutation revealed a deficiency in complex IV activity as compared with controls suggesting a direct pathogenic role for DCM [142,143]. 

## 9. Conclusions and Future Prospects

mt-tRNA mutations were common among patients with cardiomyopathy (Table 2), although the exact molecular mechanisms are not fully understood. A number of mt-tRNA mutations have been identified in the past decades. mt-tRNA pathogenic mutations have structural and functional consequences, such as affecting the tRNA structure, altering 5′ or 3′ processing of tRNAs, and leading to defects in chemical modifications. Thus, these mutations would impair the normal functions of the RCCs, thereby exacerbating the mitochondrial dysfunction that is responsible for cardiomyopathy.

The diagnosis of cardiomyopathy requires ultrastructural and enzymatic histochemical evidence due to the difficulty of proving pathogenicity by genetic mutation alone. Thus, not only a genetic approach but also pathological and enzymatic histochemical diagnosis should be used as much as possible to confirm the diagnosis of mitochondrial cardiomyopathy [144].

## Figures and Tables

**Table 1:** Integrated view of human mt-tRNA modification.

Position	Location in tRNA	Modification	Human Gene	Enzymatic Activity	Function
9	D-arm	m^1^A	*TRMT10A*	Methylation	Prevention of the Watson-Crick base pairing of A-U
9	D-arm	m^1^G	*TRMT5*	Methylation	Maintenance of the tRNA structure
10	D-arm	m^2^G	*TRMT11*	Methylation	Stabilization of overall tRNA structure
16	D-arm	m^1^A	*TRMT10A*	Methylation	Increasing the steady-state level of tRNA
20	D-arm	D	*DUS2L*	Hydrogen addition to U	Destabilization of the helical structure
26	D-arm	m^2^G	*TRMT1*	Methylation	Stabilization of tRNA tertiary structure
26	D-arm	m^2^_2_G	*TRMT1*	Methylation	Prevention of the Watson-Crick base pairing of G-C
27	Anticodon stem	Ψ	*PUS3*	Pseudouridylation	Stabilization of tRNA helical structure
28	Anticodon stem	Ψ	*PUS3*	Pseudouridylation	Enhancing the functions of tRNA
31	Anticodon stem	Ψ	*PUS3*	Pseudouridylation	Enhancing the functions of tRNA
32	Anticodon stem	m^3^C	*METTL6*	Methylation	Increasing translational output
33	Anticodon stem	Ψ	*PUS3*	Pseudouridylation	Increasing the stability of tRNA
34	Anticodon stem	τm^5^U	*MTO1, GTPBP3*	Taurinomethylation	Enable precise and efficient decoding
34	Anticodon stem	cmnm^5^U	*GTPBP3*	Taurinomethylation	Regulation of tRNA local structure
34	Anticodon stem	τm^5^s^2^U	*MTU1*	Thiolation	Regulation of tRNA local structure
34	Anticodon stem	f^5^C	*NSUN3, ALKBH1*	Methylation, Oxidization (m^5^C to f^5^C, m^5^Cm to hm^5^Cm to f^5^Cm)	Regulation of tRNA local structure
34	Anticodon stem	Q	*hQTRT1, QTRT1*	G to Q base swapping	Inhibition of RNase-mediated degradation
35	Anticodon stem	Ψ	Unidentified	/	/
37	Anticodon stem	m^1^G	*TRMT5*	Methylation	Stabilization of codon-anticodon pairing
37	Anticodon stem	t^6^A	*YRDC, OSGEP*	Threonylcarbamoylation of A	Increasing the base-stacking interactions
37	Anticodon stem	i^6^A	*TRIT1*	Isopentenylation of A	Enable precise and efficient decoding
37	Anticodon stem	ms^2^i^6^A	*TRIT1, CDK5RAP1*	Isopentenylation of A, Methylthiolation of i^6^A	Regulation of tRNA local structure
48	TψC loop	m^5^C	*NSUN2*	Methylation	Inhibition of angiogenin-mediated tRNA cleavage
49	TψC loop	m^5^C	*NSUN2*	Methylation	Inhibition of angiogenin-mediated tRNA cleavage
50	TψC loop	m^5^C	Unidentified	Methylation	Inhibition of angiogenin-mediated tRNA cleavage
50	TψC loop	Ψ	Unidentified	/	/
54	TψC loop	m^5^U	*TRMT2A*	Methylation	Prevention of tRNA cleavage
55	TψC loop	Ψ	Unidentified	/	/
58	TψC loop	m^1^A	*TRMT61B*	Methylation	Increasing the binding energies of T54-m^1^A58
66	Acceptor arm	Ψ	Unidentified	/	/
67	Acceptor arm	Ψ	Unidentified	/	/
68	Acceptor arm	Ψ	Unidentified	/	/

Abbreviations of tRNA modifications: m^1^A: 1-methyladenosine; m^1^G: 1-methylguanosine; m^2^G: *N^2^*-methylguanosine; D: dihydrouridine; m^2^_2_G: *N^2^,N^2^*-dimethylguanosine; Ψ: pseudouridine; m^3^C: 3-methylcytidine; τm^5^U: 5-taurinomethyluridine; cmnm^5^U: 5-carboxymethylaminomethyl; τm^5^s^2^U: 5-taurinomethyl-2-thiouridine; f^5^C: 5-formylcytidine; Q: queuosine; t^6^A: *N^6^*-threonylcarbamoyladenosine; i^6^A: *N^6^*-isopentenyladenosine; ms^2^i^6^A: 2-methylthio-*N^6^*-isopentenyladenosine; m^5^C: 5-methylcytidine; m^5^U: 5-methyluridine.

**Table 2:** Summary of cardiomyopathy-associated mt-tRNA mutations.

tRNA Species	Mutation	Position	Structural Location	Homoplasmy/Heteroplasmy	Aberrant tRNA Biology	Clinical Diseases	References
tRNA^Phe^	T593C	17	D-arm	Homoplasmy	Reduced expression of functional tRNA	Cardiomyopathy, optic neuropathy, and cognitive disability	[68]
tRNA^Val^	C1628T	27	Anticodon stem	Heteroplasmy	Reduce the steady-state level of tRNA	Cardiomyopathy, external ophthalmoplegia, and pigmentary retinitis	[72]
tRNA^Val^	G1644A	43	Variable region	Heteroplasmy	Reduce the steady-state level of tRNA	Cardiomyopathy, loss of balance, and progressive encephalopathy	[72]
tRNA^Leu(UUR)^	A3243G	14	D-arm	Heteroplasmy	Affect steady-state level and tRNA modification	Cardiomyopathy, MELAS-like syndrome, MERRF-like syndrome, MIDD	[73,74,75,82,83,84,85]
tRNA^Leu(UUR)^	T3250C	21	D-arm	Heteroplasmy	Affect OXPHOS functions	Cardiomyopathy, mitochondrial myopathy, and exercise intolerance	[76]
tRNA^Leu(UUR)^	A3260G	31	Anticodon stem	Heteroplasmy	Affect OXPHOS functions	Maternally inheritedmyopathy and cardiomyopathy, heart failure, MELAS-like syndrome	[77,78]
tRNA^Leu(UUR)^	T3271C	39	Anticodon stem	Homoplasmy	Disrupt conserved base pairing	Hypertrophic cardiomyopathy	[79,97]
tRNA^Leu(UUR)^	C3303T	72	Acceptor arm	Heteroplasmy	Affect CCA addition	Maternally inherited myopathy and cardiomyopathy	[80]
tRNA^Ile^	T4277C	15	D-arm	Homoplasmy	Affect tRNA steady-state level	Hypertrophic cardiomyopathy, hearing impairment	[104]
tRNA^Ile^	A4295G	37	Anticodon stem	Heteroplasmy	Affect tRNA modification	Hypertrophic cardiomyopathy, hearing impairment	[105]
tRNA^Ile^	A4300G	42	Anticodon stem	Heteroplasmy	Affect steady-state level of tRNA	Maternally inherited cardiomyopathy	[108]
tRNA^Ile^	A4317G	59	TψC loop	Homoplasmy	Affect CCA addition	Fatal infantile cardiomyopathy, deafness	[111]
tRNA^Ile^	4322dupC	64	TψC loop	Heteroplasmy	Disrupt conserved base pairing	Idiopathic dilated cardiomyopathy	[113]
tRNA^Trp^	G5521A	10	D-arm	Homoplasmy	Disrupt conserved base pairing	Maternally inherited myopathy and cardiomyopathy	[114]
tRNA^Cys^	A5814G	13	D-arm	Heteroplasmy/Homoplasmy	Disrupt conserved base pairing	Maternally inherited myopathy and cardiomyopathy	[115,116]
tRNA^Ser(UCN)^	A7495G	20	D-arm	Heteroplasmy	Disrupt conserved base pairing	Developmental delay, epilepsy, and cardiomyopathy	[118]
tRNA^Lys^	T8306C	12	D-arm	Heteroplasmy	Disrupt conserved base pairing	Myopathy, myoclonus, leukoencephalopathy, hearing loss, hypertrophic cardiomyopathy, and insulin resistance	[119]
tRNA^Lys^	A8344G	53	TψC loop	Heteroplasmy	Defect in tRNA modification	MERRF-like syndrome, cardiomyopathy, Leigh syndrome	[123]
tRNA^Lys^	G8363A	72	Acceptor arm	Heteroplasmy	Affect the 3′ end processing	MERRF-like syndrome, cardiomyopathy, and Leigh syndrome	[127,128]
tRNA^Gly^	T9997C	7	Acceptor arm	Homoplasmy	Affect the 5′ end processing	Hypertrophic cardiomyopathy	[131]
tRNA^His^	G12192A	42	TψC loop	Homoplasmy	Disrupt conserved base pairing	LHON, hearing loss, and cardiomyopathy	[133,134]
tRNA^Leu(CUN)^	T12297C	31	Anticodon stem	Heteroplasmy	Disrupt conserved base pairing	Dilated cardiomyopathy	[135,136]
tRNA^Glu^	T14709C	36	Anticodon stem	Heteroplasmy	Affect OXPHOS functions	Infantile cardiomyopathy, sensorineural hearing loss, and seizures	[141]
tRNA^Thr^	A15924G	37	Anticodon stem	Homoplasmy	Disrupt conserved base pairing	Dilated cardiomyopathy	[142]

Abbreviations: OXPHOS: oxidative phosphorylation; MELAS: mitochondrial myopathy, encephalopathy, lactic acidosis, and stroke-like episodes; MIDD: maternally inherited diabetes and deafness; MERRF: myoclonus, epilepsy, and ragged-red-fibers; LHON: Leber’s hereditary optic neuropathy.

## Data Availability

Not applicable.
